# An Impedance Aptasensor with Microfluidic Chips for Specific Detection of H5N1 Avian Influenza Virus

**DOI:** 10.3390/s150818565

**Published:** 2015-07-29

**Authors:** Jacob Lum, Ronghui Wang, Billy Hargis, Steve Tung, Walter Bottje, Huaguang Lu, Yanbin Li

**Affiliations:** 1 Cell and Molecular Biology Program, University of Arkansas, Fayetteville, AR 72701, USA; E-Mail: jlum@pacificvetgroup.com; 2 Department of Biological and Agricultural Engineering, University of Arkansas, Fayetteville, AR 72701, USA; E-Mail: rwang@uark.edu; 3 Department of Poultry Science, University of Arkansas, Fayetteville, AR 72701, USA; E-Mails: bhargis@uark.edu (B.H.); wbottje@uark.edu (W.B.); 4 Department of Mechanical Engineering, University of Arkansas, Fayetteville, AR 72701, USA; E-Mail: chstung@uark.edu; 5 Animal Diagnostic Laboratory, Pennsylvania State University, University Park, PA 16802, USA; E-Mail: hxl15@psu.edu

**Keywords:** impedance aptasensor, avian influenza virus, aptamer, microfluidic chip, virus detection

## Abstract

In this research a DNA aptamer, which was selected through SELEX (systematic evolution of ligands by exponential enrichment) to be specific against the H5N1 subtype of the avian influenza virus (AIV), was used as an alternative reagent to monoclonal antibodies in an impedance biosensor utilizing a microfluidics flow cell and an interdigitated microelectrode for the specific detection of H5N1 AIV. The gold surface of the interdigitated microelectrode embedded in a microfluidics flow cell was modified using streptavidin. The biotinylated aptamer against H5N1 was then immobilized on the electrode surface using biotin–streptavidin binding. The target virus was captured on the microelectrode surface, causing an increase in impedance magnitude. The aptasensor had a detection time of 30 min with a detection limit of 0.0128 hemagglutinin units (HAU). Scanning electron microscopy confirmed the binding of the target virus onto the electrode surface. The DNA aptamer was specific to H5N1 and had no cross-reaction to other subtypes of AIV (e.g., H1N1, H2N2, H7N2). The newly developed aptasensor offers a portable, rapid, low-cost alternative to current methods with the same sensitivity and specificity.

## 1. Introduction

The H5N1 subtype of the avian influenza virus (AIV) has caused the most lethal outbreaks of highly pathogenic avian influenza (HPAI) in poultry and fatal infections in human cases for over a decade. The H5N1 HPAI outbreaks occurred initially in Southeast Asian countries in the late 1990s but soon spread to Middle Eastern and European countries. The poultry industry has lost millions to billions of dollars in revenue in the H5N1-affected countries or regions [1], and, more importantly, the H5N1 AIV has remained a threat to human health due to its ability to mutate or recombine with other subtypes to become a lethal human pathogen. Since 2003, a total of 784 H5N1 human cases with 429 deaths and 60% mortality have been reported [2]. Rapid and effective assays for specific H5N1 detection are essential to monitor its current infection status in poultry flocks or various avian species and prevent potential outbreaks [3]. Diagnostic tests of HPAI are usually conducted in a biosecurity level 3 (BSL-3) laboratory and can take up to several days to complete with high costs. An in-field screening test of HPAI is needed for dealing with more samples and more effective quantities.

Currently virus isolation and real-time RT-PCR are commonly used for AIV surveillance tests, but virus isolation is time-consuming, and the real-time RT-PCR equipment and reagents are expensive and require specialized facilities and well-trained personnel. Commercially available rapid detection assays such as ELISA and immunochromatographic strip tests lack the required sensitivity and specificity to compete with the gold standard methods [4]. Biosensors, which combine a target-specific biological element with a transducer and signal processing unit, have shown great promise in their applications for pathogen detection in food safety, environmental monitoring, and clinical diagnostics. Some biosensors have been reported for the detection of AIV using methods such as surface plasmon resonance [5,6,7,8,9], quartz crystal microbalance [10,11,12,13], fluorescence [14,15], optical interferometry [16], imaging ellipsometry [17], and electrochemistry [18,19,20]. Recently, Jarocka’s group developed an electrochemical immunosensor for detection of antibodies against AIV H5N1 in hen serum [21]. These developed sensors have shown potential but are not suitable for rapid in-field testing due to either lack of specificity, high complexity, consuming too much time and money, or not being practical for use on site or in field conditions.

Impedance biosensors measure changes in the electrochemistry of a sample to detect a specific analyte. They have several advantages over conventional virology methods and also other types of biosensor assays for AIV detection. More importantly, impedance biosensors can be easily miniaturized and have a low cost and simple design. Combining an impedance biosensor with an interdigitated microelectrode gives further advantages of low ohmic drop, rapid establishment of steady state, rapid reaction kinetics, increased signal-to-noise ratio, and reduced sample size and detection time due to rapid response time [22]. The addition of microfluidics to biosensors allows for precise control of small sample volumes, faster detection times due to the proximity of the sample to the transducer, and high surface area to volume ratio. The ability to work with a small sample size allows for the concentration of a larger sample resulting in more sensitive detection, and also means that the person performing the test is less exposed to potentially dangerous pathogens [23].

DNA aptamers are single-stranded oligonucleotides that can be selected to bind to specific targets such as proteins, carbohydrates, lipids, small organic and inorganic molecules, and metal ions [24,25,26,27,28]. They have been looked to as alternatives to antibodies due to a number of advantages such as high thermal and chemical stability, chemical selection that allows for a great deal of freedom in the selection pressures, and chemical synthesis, which results in low cost and no batch-to-batch variation [29,30,31]. This study also suggested that aptamers have added advantages when used in impedance biosensors in that their small size and uniformity result in low noise and high repeatability.

Several impedance biosensors have previously been developed for the detection of the AIV H5 subtype or H5N1. A non-Faradic impedance biosensor was investigated in combination with a microfluidic flow cell containing an embedded interdigitated microelectrode array and immunomagnetic separation using anti-H5 antibody-coated magnetic nanobeads. A lower detection limit of 10^3^ EID_50_·mL^−1^ was achieved and was specific for the H5 subtype [32,33]. The second non-Faradic biosensor developed for the detection of H5N1 AIV used immunomagnetic separation with anti-H5 antibody-coated magnetic nanobeads, a microfluidic flow cell with an embedded interdigitated microelectrode that was coated in anti-N1 antibody and chicken red blood cell (RBC) labels for amplification [34]. This biosensor was capable of specifically detecting H5N1 AIV at 10^3^ EID_50_·mL^−1^ but had a detection time of 2 h and required multiple steps in the detection protocol. A Faradic impedance biosensor was developed using an open interdigitated microelectrode array with immobilized polyclonal antibody against H5, and when RBC amplification was used the sensor had a lower detection limit of 10^3^ EID_50_·mL^−1^ [35].

In our previous research studies, several aptamer-based biosensors were developed for AIV detection, such as an SPR aptasensor [6] and QCM aptasensors [13,36]. Though all of these aptasensors were specific and sensitive, the SPR aptasensor had a relatively long detection time (1.5 h), and the QCM aptasensors were not practical for in-field use due to the QCM’s predisposition to environmental noise [37]. Recently, an impedance-based aptasensor was developed for the detection of H5N1 AIV with enhanced sensitivity [38]. However, this reported method required signal amplification with labels, and the detection time was prolonged to 2 h. In this study, a simple design of the aptasensor with impedance measurement was developed, which made it a prime candidate for miniaturization and in-field use. It overcame several disadvantages from the previous biosensors, namely long electrode preparation time and reliance on nanoparticles or biolabels.

## 2. Experimental Section

### 2.1. Materials

Aptamers specific against H5N1 AIV were developed in our group with detailed information described in our previous study [39,40]. Selection and characterization of DNA aptamers were carried out using Systematic Evolution of Ligands by EXponential enrichment (SELEX) technology and SPR. The best aptamer had a dissociation constant (*K_D_*) of 4.65 nM with a sequence of 5′-GTG TGC ATG GAT AGC ACG TAA CGG TGT AGT AGA TAC GTG CGG GTA GGA AGA AAG GGA AAT AGT TGT CCT GTT G-3′. The most favorable secondary structure of the best aptamer is shown in the Supplementary Materials (Structure 1 in Table S1). The secondary structure of the aptamer was predicted by web-based UNAFold software using the OligoAnalyzer 3.1 program from IDT (Integrated DNA Technologies, Coralville, IA, USA), which was based on a free energy minimization algorithm. The ∆G value for the aptamer structure was −7.03 kcal/mol. Other predicted secondary structures are also listed in Table S1 in the Supplementary Materials. Biotinylated aptamers were synthesized by Integrated DNA Technologies (Coralville, IA, USA) with biotin conjugated at the 5′-end. They were reconstituted in phosphate-buffered saline (PBS) to a concentration of 220 μg·mL^−1^. A control sequence of 5′-CCG AAT TCG AAG GAC AAG AGG CGA AAA GAT TTA AAG TAA TCA AAG ACT GAG CAA CTC TTA TCT TTT ATG CTA CGT CCC GC-3′ was used for the control test. The PBS (10 mM, pH 7.4) was purchased from Sigma-Aldrich (St. Louis, MO, USA). The washing solution (0.04 M imidazole buffered saline with 0.4% Tween 20) was purchased from KPL, Inc. (Gaithersburg, MD, USA) and diluted with Milli-Q water (18.2 MΩ cm, Millipore, Bedford, MA, USA) to a 1:200,000 dilution for use as a measuring buffer. Streptavidin was purchased from Rockland Immunochemicals, Inc. (Gilbertsville, PA, USA) and reconstituted in PBS to a concentration of 0.2 mg·mL^−1^. Inactivated H5N1 AIV (Scotland/59) was provided by the APHIS-USDA National Veterinary Services Laboratories (NVSL, Ames, IA, USA). The stock virus titer of H5N1 AIV was 128 hemagglutination units (HAU) 50 μL^−1^. Hemagglutination (HA) is defined as the agglutination of chicken red blood cells that is caused by the presence of a hemagglutinin virus. One HA unit (HAU) in a virus suspension is the minimum amount of virus that will cause complete agglutination of the red blood cells [6], or one HAU in a virus suspension is measured by the amount of virus dilutions made equal to the amount of HA titers. Other subtypes of H7N2, H1N1, and H2N2 AIVs were provided by the Penn State Animal Diagnostics Laboratory (University Park, PA, USA). All viruses used in this study were inactivated by the providers using β-propiolactone, eliminating infectivity while preserving HA activity [41]. Sterile PBS was used for virus dilutions.

### 2.2. Microfluidics Biochips with Embedded Interdigitated Microelectrodes

A microfluidics biochip (shown in Figure S1, Supplementary Materials) with an embedded gold interdigitated microelectrode was designed and fabricated using the method described by Varshney *et al.* [34] with two important improvements. First, a microfluidic channel (40 μm deep and 100 μm wide) with an oval-shaped microfluidics chamber (40 μm deep, 500 μm wide, and 1723 μm long; 34.5 nL volume) was designed to replace the square-shaped chamber used in our previous study [42], which could minimize the residues retained at the corner of the square chamber during the washing step. The microfluidic channel was molded from polydimethylsiloxane (PDMS) and fixed to an interdigitated microelectrode chip with a glass substrate. Second, the width of electrode fingers was reduced from 25 to 10 μm, since the small-scale electrode fingers could result in improved sensitivity [43]. Each electrode consisted of 25 pairs of 10 μm wide electrode fingers spaced 10 μm apart.

### 2.3. Aptamer Immobilization

The experimental protocol consisted of the immobilization of a specific aptamer onto the microelectrode surface followed by the capture of influenza virus and impedance measurement, shown in Figure S2. After each immobilization/capture step, the microfluidic chip was washed with measuring buffer for 2 min at a rate of 16.7 μL·min^−1^ to remove any unbound particles. The pump was then stopped and the impedance was measured after a 2-min incubation period. All incubations and measurements were done at ambient temperature (18–24 °C).

The microfluidic chip was cleaned by pumping Milli-Q water (Milli-Q, 18.2 MΩ cm, Bedford, MA, USA) for 15 min at a rate of 16.7 μL·min^−1^. Streptavidin (0.2 mg·mL^−1^) was injected into the microfluidic chip at a flow rate of 16.7 μL·min^−1^ and then the pumping was stopped to allow for a 30 min incubation period. The streptavidin was immobilized through direct physical adsorption onto the gold electrode. Forces involved in the adsorption process might include Van der Waals forces, ionic bonds, and/or hydrogen bonds. A botin-labeled aptamer specific for H5N1 AIV was injected and incubated for 30 min, allowing the aptamer to be immobilized through streptavidin–biotin binding (*K_d_* = 10^−14^ M) [44].

### 2.4. AIV Detection

Impedance measurements were taken using an IM-6 impedance analyzer with IM-6/Thales 2.49 software (BAS, West Lafayette, IN, USA). The wires connected to the microfluidic chip were attached to the test-sense and counter-reference probes of the impedance analyzer. A sinusoidal AC potential of 100 mV was applied for all impedance measurements. The 100 mV potential was used in the study to overcome noise while the impedance was still linearly measured [42]. Impedance magnitude and phase angle were measured at 54 points in the frequency range from 1 Hz to 1 MHz. All impedance measurements were done in the presence of a measuring buffer.

A virus sample was injected into the microfluidic flow cell and incubated for 30 min. After washing, the impedance was measured. The washing step helped to remove any extraneous material that might be present in an actual sample as well as any material that might have nonspecific effects on impedance. The impedance change was calculated as the virus impedance minus the impedance of the aptamer immobilization. Ten-fold serial dilutions of the H5N1 AIV, measured from 12.8 to 0.00128 in HAU, were prepared for the impedance measurements. Triplicate tests were conducted at each virus dilution to determine the effect of virus concentration on the impedance change and to form a calibration curve for the sensor. A PBS sample without virus was used as a negative control. Non-target AIV subtypes of H1N1 and H2N2 were used to determine that there was no cross-reaction or specificity of the aptasensor.

### 2.5. Electron Microscopy

Environmental scanning electron microscopy (ESEM) was used to confirm the binding of AIV onto the electrode surface. Samples for ESEM were prepared by modifying the electrode surface with an aptamer against H5N1 as described in Section 2.3. An H5N1 virus suspension at a concentration of 12.8 HAU was injected onto the electrode surface and incubated for 30 min. Sterile deionized water was then pumped into the flow cell at a rate of 16.7 μL·min^−1^ for 10 min to remove excess salts from the sample. Thereafter, the PDMS flow cell was removed from the electrode and the sample was allowed to dry in a fume hood overnight. No critical point of drying or sputter coating was needed to prepare the samples. A Philips FEI XL-30 environmental scanning electron microscope (FEI, Hillsboro, OR, USA) was used to take electron micrographs under a vacuum.

### 2.6. Statistical Analysis

Microsoft Excel (Microsoft, Redmond, VA, USA) was used for statistical analysis of all data and graphs preparation. Mean values and standard deviations were calculated based on triplicate tests for each dilution or concentration of the test virus HAU. Statistically significant differences were determined using *t*-tests (α = 0.05).

## 3. Results and Discussion

### 3.1. Characterization of Impedance Data

**Figure 1:**
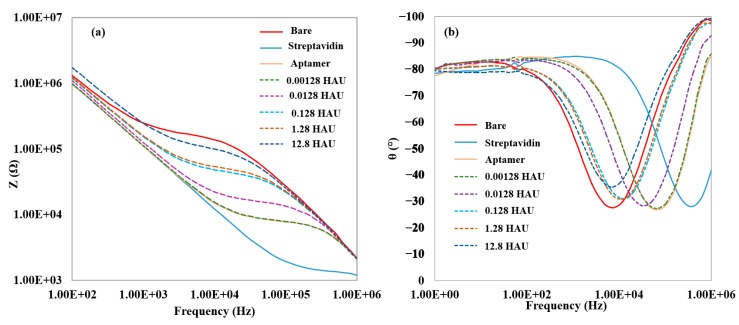
(**a**) Typical impedance magnitude data for the detections of H5N1 AIV; (**b**) Typical phase angle data for the detection of H5N1 AIV. Data labels for dashed lines correspond to serial dilution values (10^1^ to 10^5^ dilutions) of 128 HAU virus sample (12.8 to 0.00128 HAU). The amplitude of voltage was 100 mV.

Figure 1a shows the impedance magnitude at each step of the aptamer immobilization and H5N1 AIV detection by the aptasensor. The physical adsorption of streptavidin onto the electrode surface caused a large decrease in impedance as compared to the pure measuring buffer. The reason for the decrease in impedance value after streptavidin binding was likely due to a shift in the isoelectric point at the electrode surface, resulting in the local ion concentration being increased. This had the effect of decreasing the solution resistance and increasing the capacitance in the biosensor, thus decreasing the impedance value [45]. An increase in impedance was seen after incubation with aptamers, indicating successful immobilization of aptamers through biotin-streptavidin binding. The capture of AIV onto the modified electrode surface further increased the impedance, with the increase in impedance correlating to the virus concentration. The impedance increase caused by the binding of the virus was likely caused by the blockage of the flow of ions between the electrode fingers. Figure 1b shows the phase angle data for each step of the aptamer immobilization and H5N1 AIV detection by the aptasensor. The phase angle describes the relative contributions of the real and imaginary elements to the total impedance value. A phase angle of −90° is the result of a purely capacitive system, whereas a phase angle of 0° is the product of a system that is purely resistance. The dip in the phase angle in the mid-frequency range indicates where the capacitive portion of impedance contributes the least to the impedance measurement. For the virus detection, the phase angle is close to −90° on both ends of the impedance spectrum, while it approaches −25° to −30° in the middle frequency range. From this, it can be concluded that the capacitive portion of impedance is dominant at the high and low ends of the frequency range while the resistance dominates the mid-frequency range, where the largest amount of impedance magnitude change is seen. This is consistent with the previous biosensor design using a microfluidic biochip [34]. At the frequency at which the greatest amount of impedance magnitude is seen (25.8 kHz, determined by percent change), the phase angle approaches −25° to −30°, suggesting that the real part of the impedance plays a major role in the impedance change as compared to the imaginary part.

**Figure 2:**
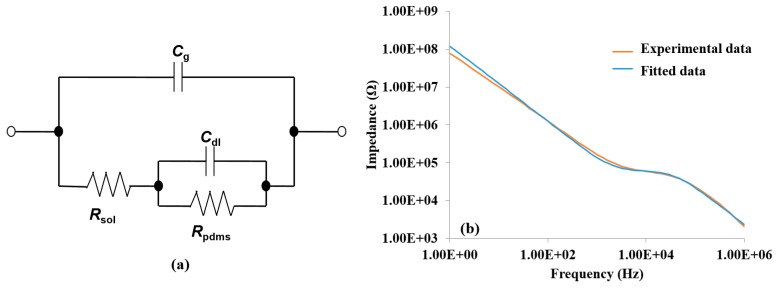
(**a**) The equivalent circuit used for data analysis. The equivalent circuit components were resistance of the solution (*R*_s_), resistance of PDMS (*R*_pdms_*)*, double layer capacitance (*C*_dl_), and geometrical capacitance (*C*_g_); (**b**) The bode diagram of measured impedance data and simulated impedance data generated by curve fitting of the equivalent circuit. The diagram data was measured by testing a H5N1 virus with a concentration of 12.8 HAU.

The roles of the real and imaginary parts were confirmed by constructing an equivalent circuit model, as shown in Figure 2a. The circuit consisted of two resistor elements, *R_sol_* and *R_pdms_*, and two capacitive elements, *C_g_* and *C_dl_*. The resistor elements corresponded to the resistance of the electrolyte solution (*R_sol_*) and the resistance of the PDMS layer connecting the electrode fingers (*R_pdms_*), while *C_g_* and *C_dl_* corresponded to the geometric capacitance of the electrolyte solution and the double layer capacitor formed by the ions near the electrode surface, respectively. This equivalent circuit was found to hold true for H5N1 detection. Data collected from the detection of 12.8 HAU for H5N1 AIV was used for fitting analysis. Fifty-four points from the experimental data were chosen by the software to fit a simulated impedance spectrum. The mean error between the experimental and simulated spectrums was 2.1% for the impedance magnitude and 0.9° for the phase angle, while the maximum error was 12.9% for impedance magnitude and 13° for the phase angle. A bode plot of the curve fitting analysis is shown in Figure 2b. The mean error between the experimental and simulated spectrums for other concentrations (1.28, 0.128, and 0.0128) were 2.7%, 1.8%, and 2.3% for the impedance magnitude, respectively, and 0.8°, 1.2°, and 0.9° for the phase angle, respectively.

The role of each equivalent circuit element was further investigated to understand the phenomenon causing the impedance changes. Two elements were found to contribute significantly to the impedance change, *R_sol_* and *C_dl_*. The values of the elements of the equivalent circuit, as determined from the curve fitting analysis, are shown in Table 1.

**Table 1:** Contributions of the elements in the equivalent circuit to the impedance magnitude. Impedance magnitude values were calculated using simulated data from fitting the equivalent circuit to measured data gathered in the detection of 12.8 HAU for H5N1 AIV.

	*R*_sol_ (kΩ)	*R*_pdms_ (kΩ)	*C*_dl_ (nF)	*C*_g_ (nF)
Bare electrode	187.3	0.271	1.269	0.052
Streptavidin	1.4	0.250	1.462	0.089
Aptamer	9.4	0.282	1.486	0.074
Virus	64.1	0.388	0.870	0.066
% of change between aptamer and virus	582	37.6	−41.5	−10.3
*p*-value between aptamer and virus	<0.01	0.20	0.02	0.32

When using the simulated values to calculate the individual element contributions to the impedance change when measuring a H5N1 AIV sample at 12.8 HAU, the *C_dl_* element accounted for only 1 kΩ (1.8%) of the total impedance magnitude change, while the *R_sol_* element accounted for 54.7 kΩ (98.2%) of the impedance magnitude change. The *R_pdms_* and *C_g_* element contributions to the impedance magnitude change were negligible. The *R_sol_* and *C_dl_* contributions to the impedance magnitude change confirm what was suggested by the phase angle data, namely that the real part, specifically *R_sol_*, dominates at the frequency at which the greatest impedance magnitude change is seen. The *R_sol_* value had a large decrease after the addition of streptavidin, likely due to a shift in the isoelectric point as described previously. The aptamer binding increased the *R_sol_* value. This increase may have been due to the change in the electrochemical environment of the electrode surface created by the negatively charged aptamers. The capture of virus caused a large increase in the *R_sol_* value, probably due to the physical blocking of ion flow due to the virus particle size and composition, as well as its lipid membrane acting as an insulator. Due to the importance of *R_sol_* to the impedance magnitude change, it can be assumed that the largest factor in the detection of AIV was the flow of ions between the electrode fingers, which was obstructed by the capture of virus onto the electrode surface [46]. The capture of a virus onto the electrode surface also affects the ability to form a double layer capacitor on the surface of the electrode, resulting in a small but non-negligible change in the impedance magnitude.

A control test was carried out using a non-target aptamer with the same immobilization method, and then applied for H5N1 AIV detection (12.8 HAU). Only negligible impedance increase (282 ± 121 Ω) was observed, when compared to the same case of using a target specific aptamer (61,000 ± 381 Ω).

### 3.2. Detection of H5N1 AIV

**Figure 3:**
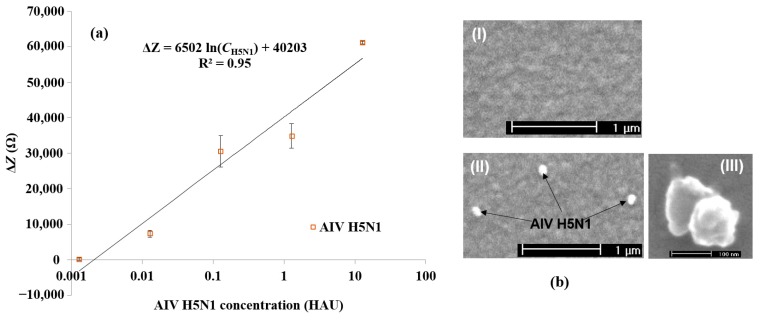
(**a**) Average impedance change caused by different concentrations of AIV H5N1. The values of the horizontal axis correspond to serial dilution values of 128 HAU virus sample (12.8–0.00128 HAU). Error bars are based on the standard deviation of triplicate tests. The impedance was measured at the frequency of 25.8 kHz; (**b**) ESEM micrographs of the electrode surface with immobilized aptamers (I) before and (II,III) after AIV capture.

Figure 3a shows the impedance magnitude change at 25.8 kHz plotted for each concentration from 12.8 to 0.00128 HAU for AIV H5N1. As stated in Section 3.1, 25.8 kHz was found to be the point at which the greatest impedance magnitude change was seen for H5N1. For the detection of H5N1 AIV, a logarithmic relationship was found between the impedance change, Δ*Z* in Ω, and the virus concentration, *C*_virus_ in log(HAU), in the range of 12.8 to 0.00128 HAU (Δ*Z* = 6502 ln(*C*_H5N1_) + 40,203; *R*^2^ = 0.95) and the detection limit was determined to be 0.0128 HAU. The detection limit was set as background (blank) signal + 3 × noise, where noise was defined as the standard deviation of the negative PBS control [11].

Although the detection limit of the aptasensor was similar to previously described impedance immunosensors [34,35] for AIV detection, the developed aptasensor in this study was capable of detecting AIV and formulating a linear calibration curve without the use of labels or pre-concentration, thereby decreasing the detection time and resources needed. This new test needs only 30 min for the completion of virus detection from sample injection to impedance measurement, one quarter of the previous immunosensor detection time [34]. Due to the small size and uniformity of the aptamers, no blocking step was needed, saving time and resources. Several hypotheses have been proposed for studying why certain aptasensors do not require a blocking step, including steric hindrance due to uniform covering of the electrode surface and charge/ion changes on the electrode surfaces [47]. Though a small amount of non-specific binding was seen, it was far below the threshold of detection. The lack of a blocking step likely increased the sensitivity of the aptasensor due to the lack of noise caused by a blocking layer. The current impedance aptasensor developed in this study had a lower detection limit and shorter detection time compared to the SPR aptasensor by Bai *et al.* [6]. The QCM aptasensor developed by Wang and Li [13] had the same detection limit and detection time as this current impedance aptasensor, but the QCM aptasensor is not practical for in-field use due to the QCM’s predisposition to environmental noise [37]. A comparison study between the developed aptasensor and other methods [4,6,11,13,36,38,48,49] for AIV H5 subtype detection based on the same virus unit (HAU) is summarized in Table S2 (Supplementary Materials). This current impedance aptasensor is a more practical format for in-field testing because impedance biosensors are easily miniaturized, have low energy requirements, and can have simple designs. ESEM examination was used for further confirmation of the target virus binding to the aptamer-coated electrode surface, and the H5N1 virus particles (inactivated) captured on the electrode surface were observed. Figure 3b shows the ESEM images for the gold microelectrode surface before (I) and after (II) the binding of H5N1 viruses. It can be seen from Figure 3b (II) that three target H5N1 viruses were captured on the aptamer-coated electrode surface. The actual AIV H5N1 is a long filamentous or spherical virus with a diameter of 80–120 nm [50]. The viruses here appeared to be rod and/or spherical in shape, with a diameter in the range of 80 to 125 nm, which was close to the diameter of typical AI viruses (80–120 nm). The results confirmed the binding of target viruses onto the aptamer-coated electrode surface.

### 3.3. Specificity Study

**Figure 4:**
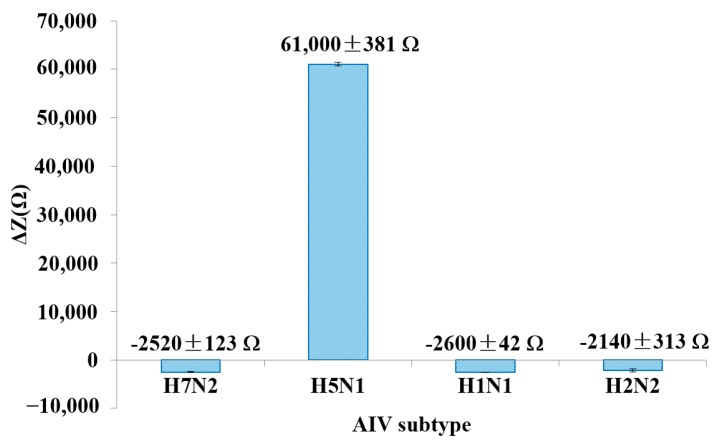
Specificity study of the developed impedance aptasensor. AIVs were tested at a concentration of 12.8 HAU. Error bars were based on the standard deviation of triplicate tests. The impedance was measured at the frequency of 25.8 kHz.

Figure 4 shows the Δ*Z* at 25.8 kHz for the detection of a target virus compared to non-target viruses at the same concentration of 12.8 HAU. The experimental data presented in Figure 4 were collected on the same day. Several chips were prepared in parallel, and then used for virus detection. Three non-target AIVs (H7N2, H1N1 and H2N2) were tested. The result showed a negative impedance change after incubation with non-target AIVs, indicating no cross interactions. The average impedance decrease for all non-target AIV tests was ~2 kΩ. The negative Δ*Z* values seen for the non-target AIVs may be due to some minor non-specific adsorption of egg proteins present in the AIV samples, namely avidin, with the gold electrode. It is known that avidin readily adsorbs onto gold surfaces and would result in the impedance decrease seen [45,51]. The specificity of the aptasensor is mainly dependent upon the aptamer that is immobilized on the electrode surface. The H5N1 aptamer used in this study was newly developed by our group, which displayed efficient binding affinity and high specificity against H5N1 AIV [39]. The stability of the aptamer surface was investigated by measuring the impedance value every hour at room temperature continuously for 8 h in the same day. An 8% decrease of impedance was observed at 8 h, indicating acceptable stability.

## 4. Conclusions

An impedance aptasensor was developed by using microfluidic flow cells with interdigitated electrodes for the rapid and specific detection of H5N1 AIV. The aptasensor was capable of detecting AIV at concentrations as low as 0.0128 HAU in 30 min. This newly developed aptasensor was capable of matching the detection limit of previously developed impedance immunosensors for AIV detection without label amplification or sample pre-concentration, while reducing the detection time and required resources. Compared to previously developed aptasensors for AIV detection, this new impedance aptasensor was equally or more sensitive but had a more practical design for in-field tests. Future research studies may focus on taking advantage of aptamers’ high stability to prepare electrodes far in advance of AIV detection (or for a long period of storage). This would make the developed aptasensor much more practical for rapid, in-field tests.

## Supplementary Files

Supplementary File 1
