# Supplementary material for: An Impedance Aptasensor with Microfluidic Chips for Specific Detection of H5N1 Avian Influenza Virus
Source: Sensors (Basel). 2015 Jul 29;15(8):18565–78. doi: 10.3390/s150818565 (PMC4570336; doi:10.3390/s150818565)
Supplement: Supplementary File 1 [file sensors-15-18565-s001.pdf]

OPEN ACCESS

sensors

ISSN 1424-8220
www.mdpi.com/journal/sensors

Supplementary Information

An Impedance Aptasensor with Microfluidic Chips for Specific Detection of H5N1 Avian Influenza Virus. *Sensors* 2015, *15*, 18565-18578

Jacob Lum [1], Ronghui Wang [2], Billy Hargis [3], Steve Tung [4], Walter Bottje [3], Huaguang Lu [5] and Yanbin Li [2,3,*]

1 Cell and Molecular Biology Program, University of Arkansas, Fayetteville, AR 72701, USA; E-Mail: jlum@pacificvetgroup.com
2 Department of Biological and Agricultural Engineering, University of Arkansas, Fayetteville, AR 72701, USA; E-Mail: rwang@uark.edu
3 Department of Poultry Science, University of Arkansas, Fayetteville, AR 72701, USA; E-Mails: bhargis@uark.edu (B.H.); wbottje@uark.edu (W.B.)
4 Department of Mechanical Engineering, University of Arkansas, Fayetteville, AR 72701, USA; E-Mail: chstung@uark.edu
5 Animal Diagnostic Laboratory, Pennsylvania State University, University Park, PA 16802, USA; E-Mail: hxl15@psu.edu

* Author to whom correspondence should be addressed; E-Mail: yanbinli@uark.edu; Tel.: +1-479-575-2881; Fax: +1-479-575-2846.

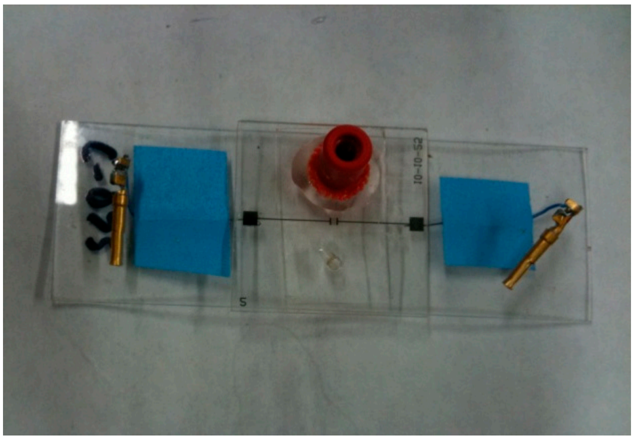

(a)

Figure S1. *Cont.*

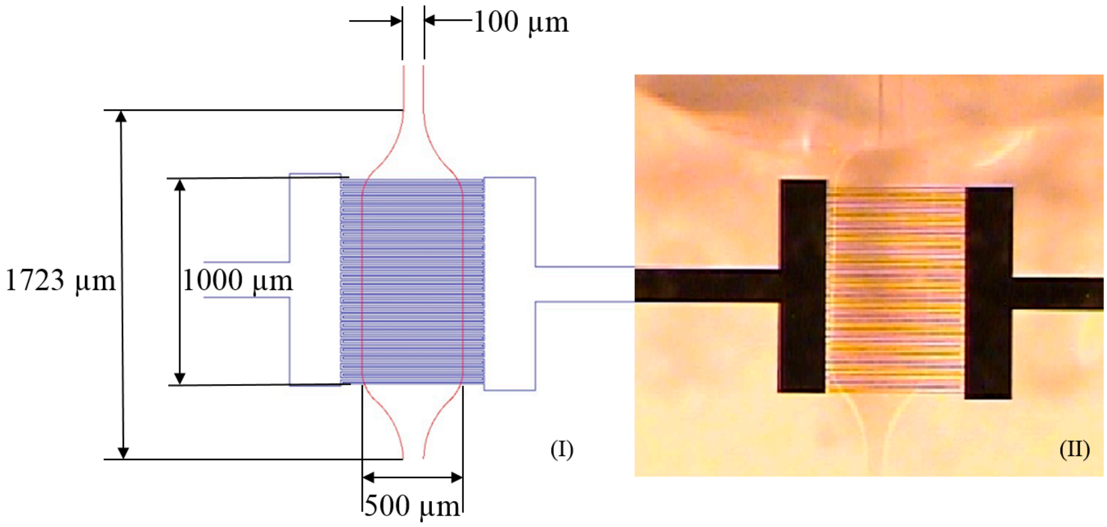

(**b**)

Figure S1. (**a**) Microfluidic chip with inlet port and embedded interdigitated microelectrode; (**b**) Gold interdigitated array microelectrode (I) drawing and (II) picture. The microfluidic channel is 40 µm deep and 100 µm wide with an oval-shaped microfluidics chamber (40 µm deep, 500 µm wide, and 1723 µm long; 34.5 nl volume). Each electrode consisted of 25 pairs of 10 µm wide electrode fingers spaced 10 µm apart.

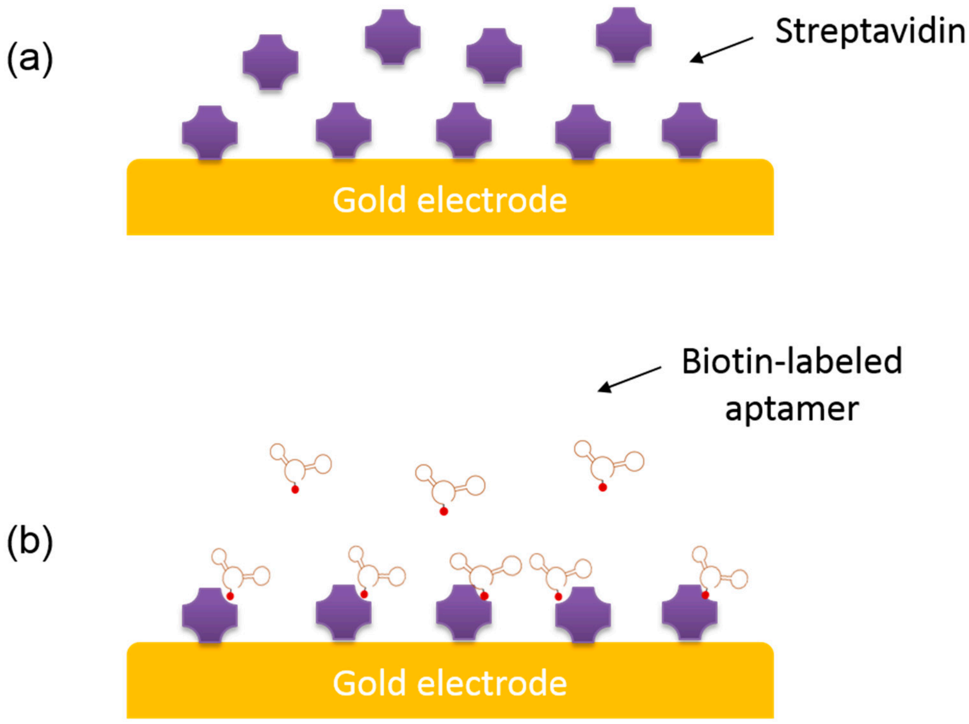

Figure S2. *Cont.*

(c)

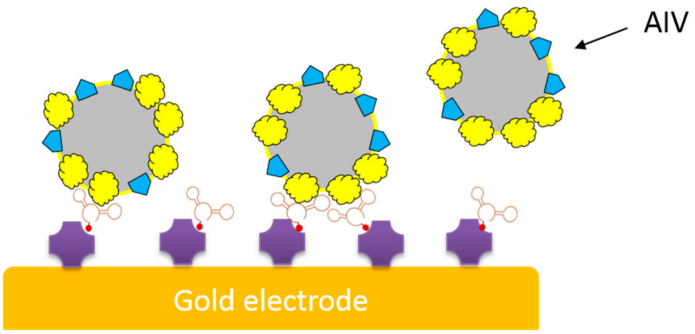

Figure S2. Design of the impedance aptasensor for the detection of AIV H5N1. (**a**) The microelectrode surface was modified using streptavidin; (**b**) The biotin-labeled aptamer was then immobilized through biotin-streptavidin binding; (**c**) Target AIV H5N1 was captured and the impedance was measured. After each step the flow cell was washed to remove unbound particles.

Table S1. Secondary structures of the H5N1 aptamer predicted by web-based UNAFold software using the OligoAnalyzer 3.1 program from IDT (Integrated DNA Technologies).

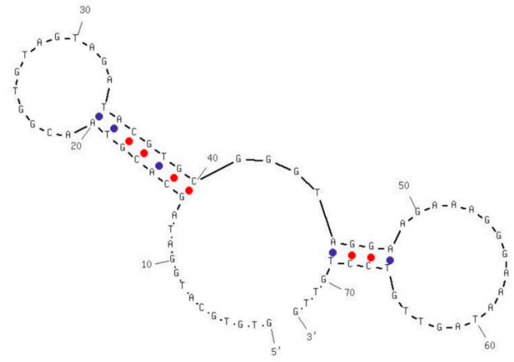 **Structure 1**: ΔG = −7.03 kcal/mol	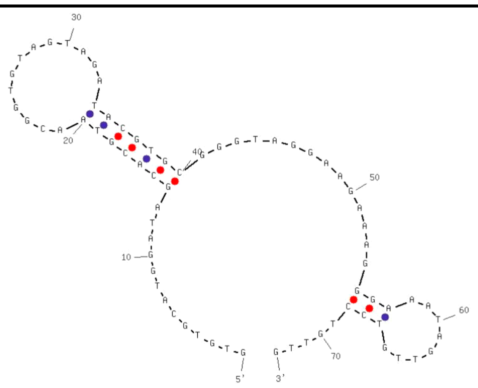 **Structure 2**: ΔG = −6.43 kcal/mol
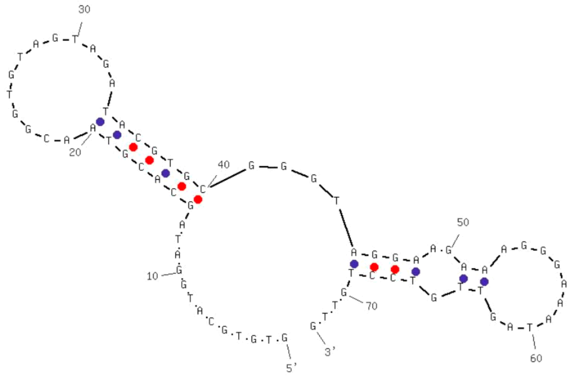 **Structure 3**: ΔG = −6.05 kcal/mol	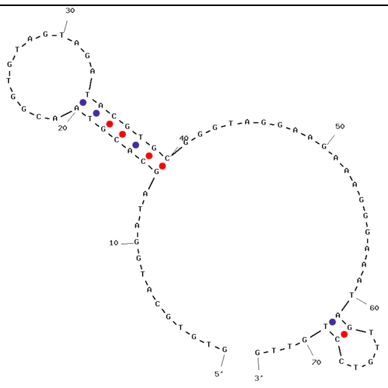 **Structure 4**: ΔG = −5.68 kcal/mol

Table S1. *Cont.*

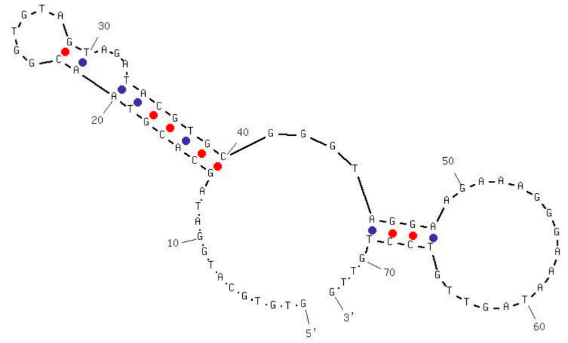 **Structure 5**: ΔG = −5.45 kcal/mol	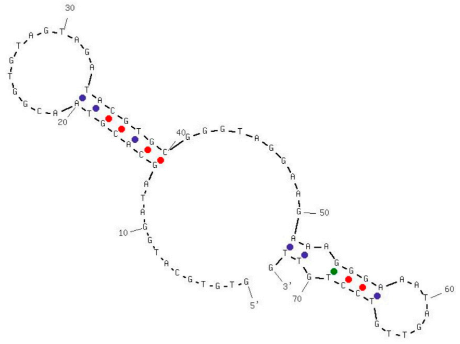 **Structure 6**: ΔG = −5.26 kcal/mol
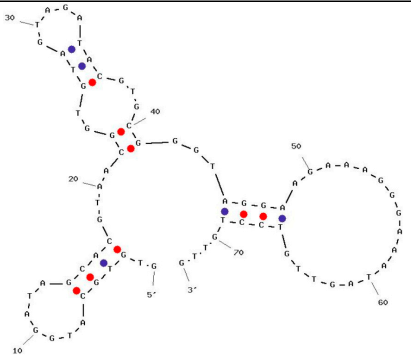 **Structure 7**: ΔG = −4.69 kcal/mol	

Table S2. A comparison study between the developed aptasensor and other methods for AIV H5 subtype detection based on the same virus unit (HAU).

Methods	Detection Time	Detection Limit	Label-Free Detection	Reference
SPR aptasensor	1.5 h	0.128 HAU	Yes	6
Hydrogel QCM aptasensor	0.5 h	0.0128 HAU	Yes	13
QCM aptasensor	1 h	1 HAU	Yes	36
QCM immunosensor	2 h	0.0128 HAU	No	11
Impedance aptasensor	2 h	0.0008 HAU	No	38
Impedance immunosensor	1 h	0.5 HAU	Yes	48
Bio-nanogate based biosensor	1 h	0.00195 HAU	Yes	49
RT-PCR	3 h	0.0256 HAU	Yes	4
Impedance aptasensor	0.5 h	0.0128 HAU	Yes	This study

© 2015 by the authors; licensee MDPI, Basel, Switzerland. This article is an open access article distributed under the terms and conditions of the Creative Commons Attribution license (http://creativecommons.org/licenses/by/4.0/).
